# Protocol for a Pragmatic Trial of Pharmacotherapy Options Following Unsatisfactory Initial Treatment in OCD (PROCEED)

**DOI:** 10.3389/fpsyt.2022.822976

**Published:** 2022-05-16

**Authors:** Pei Wang, Wenjie Gu, Jian Gao, Changhong Wang, Jianqun Fang, Maorong Hu, Hui Xiang, Bin Li, Na Liu, Wenxin Tang, Xijin Wang, Yanbin Jia, Yi Li, Yuqi Cheng, Zhen Tang, Helen Blair Simpson, Dan J. Stein, Zhen Wang

**Affiliations:** ^1^Shanghai Mental Health Center, Shanghai Jiao Tong University School of Medicine, Shanghai, China; ^2^Department of Psychiatry, The Second Affiliated Hospital of Xinxiang Medical University, Henan, China; ^3^Mental Health Center, General Hospital of Ningxia Medical University, Ningxia, China; ^4^Psychiatry Department, The First Affiliated Hospital of Nanchang University, Jiangxi, China; ^5^Guizhou Provincial People's Hospital, Guizhou, China; ^6^Mental Health Center, West China Hospital, The Sichuan University, Sichuan, China; ^7^Department of Medical Psychology, The Affiliated Brain Hospital of Nanjing Medical University, Nanjing, China; ^8^Hangzhou Seventh People's Hospital, Mental Health Center Zhejiang University School of Medicine, Zhejiang, China; ^9^The First Psychiatric Hospital of Harbin, Heilongjiang, China; ^10^Department of Psychiatry, The First Affiliated Hospital of Jinan University, Guangzhou, China; ^11^Wuhan Mental Health Center, Hubei, China; ^12^First Affiliated Hospital of Kunming Medical University, Kunming, China; ^13^Suzhou Guangji Hospital, Jiangsu, China; ^14^Department of Psychiatry, Columbia University Vagelos College of Physicians and Surgeons, New York, NY, United States; ^15^Department of Psychiatry and South African Medical Research Council Unit on Risk & Resilience in Mental Disorders, University of Cape Town, Cape Town, South Africa; ^16^Institute of Psychological and Behavioral Science, Shanghai Jiao Tong University, Shanghai, China

**Keywords:** obsessive-compulsive disorder, treatment-naïve, pharmacotherapy, alternatives, remission

## Abstract

**Background:**

Selective serotonin reuptake inhibitors (SSRIs) are the first-line pharmacotherapy for obsessive-compulsive disorder (OCD), but a large proportion of patients do not achieve remission after an adequate SSRI trial. To the best of our knowledge, there have been no well-powered randomized controlled trials (RCTs) of sequenced pharmacotherapy using pragmatic research designs. China provides a unique context for undertaking such a trial that will recruit the largest treatment-naïve participants and systematically compare the efficacy of different sequenced pharmacotherapy.

**Methods:**

A pragmatic research design will be adopted, with *n* = 1,600 treatment-naïve OCD patients initially treated for sertraline for 12 weeks, and with non-remitters then randomized to 5 different augmentation or switching pharmacotherapy options for another 12 weeks. The 5 arms will include: (1) treatment with higher than usual doses of sertraline, (2) switch to fluvoxamine, (3) switch to venlafaxine, (4) augmentation with memantine, and (5) augmentation with aripiprazole.

**Discussion:**

China is uniquely positioned to recruit sufficiently large sample sizes of treatment-naïve OCD patients to compare different pharmacotherapy options; data from the proposed trial promises to help inform current clinical practice guidelines by providing important information about optimal pharmacotherapy choice for those who demonstrate no response or response but no remission to first line pharmacotherapy.

**Trial Registration:**

The trail was registered on 27 August 2020 in ClinicalTrials.gov (https://register.clinicaltrials.gov/) (NCT04539951).

## Introduction

Obsessive-compulsive disorder (OCD) is a common and disabling disorder that has a 12-month and lifetime prevalence of 1–3% worldwide, and that is accompanied by significant morbidity, impairment and huge economic burden (1–4). OCD is characterized by obsessions and compulsions; obsessions are comprised of unwanted, intrusive and persistent thoughts and images, while compulsions are comprised of repetitive behaviors, often executed with the purpose of relieving anxiety and distress caused by obsessions (5). There is a strong evidence-base demonstrating the efficacy and tolerability of selective serotonin reuptake inhibitors (SSRIs) in OCD, and these agents are therefore viewed as a first-line pharmacotherapy in OCD treatment guidelines (6–9).

Nevertheless, a large proportion of OCD patients are response only partially or not at all to an adequate trial of an SSRI (10–12). The literature on randomized controlled trials (RCTs) of pharmacotherapy approaches to OCD when response to an SSRI is unsatisfactory is relatively sparse, with little attention to pragmatic or “real-world” research designs, or to those who respond but do not remit to treatment. Pragmatic trials are important for obtaining data on representative samples (13), while remission entails improvements in both symptoms and function and so is an important goal for patients (14–16).

On the basis of the existing sparse literature, several pharmacotherapy options for OCD patients who do not respond, or who respond but do not remit, have been outlined in current treatment guidelines. These include (1) treatment with higher than usual doses of an SSRI, (2) switch to a different SSRI, (3) switch to a different class of medication, (4) augmentation with a dopamine blocker, and (5) augmentation with a glutamatergic agent. There is a need for additional data, particularly real-world data, on how best to choose between these options.

China provides a unique context for undertaking appropriately powered RCTs to compare different pharmacotherapy options for those who do not remit following an adequate SSRI initial trial. Our research will be able to recruit the largest treatment-naïve sample in a clinical trial to date, and such data may be extremely useful for informing evidence-based treatment guidelines on optimal pharmacotherapy of treatment-naïve OCD. The proposed RCT will adopt a pragmatic treatment design, recruiting OCD patients for a 12-week trial of sertraline, and then randomizing non-remitters to 5 different treatment options.

## Methods

### Design

This study is a multi-center clinical study with a total of 13 clinical centers that specialize in the management of patients with OCD. In the Chinese context, such centers evaluate and manage many treatment-naïve patients. This allows such centers to undertake well-powered pragmatic research trials.

**Figure 1:**
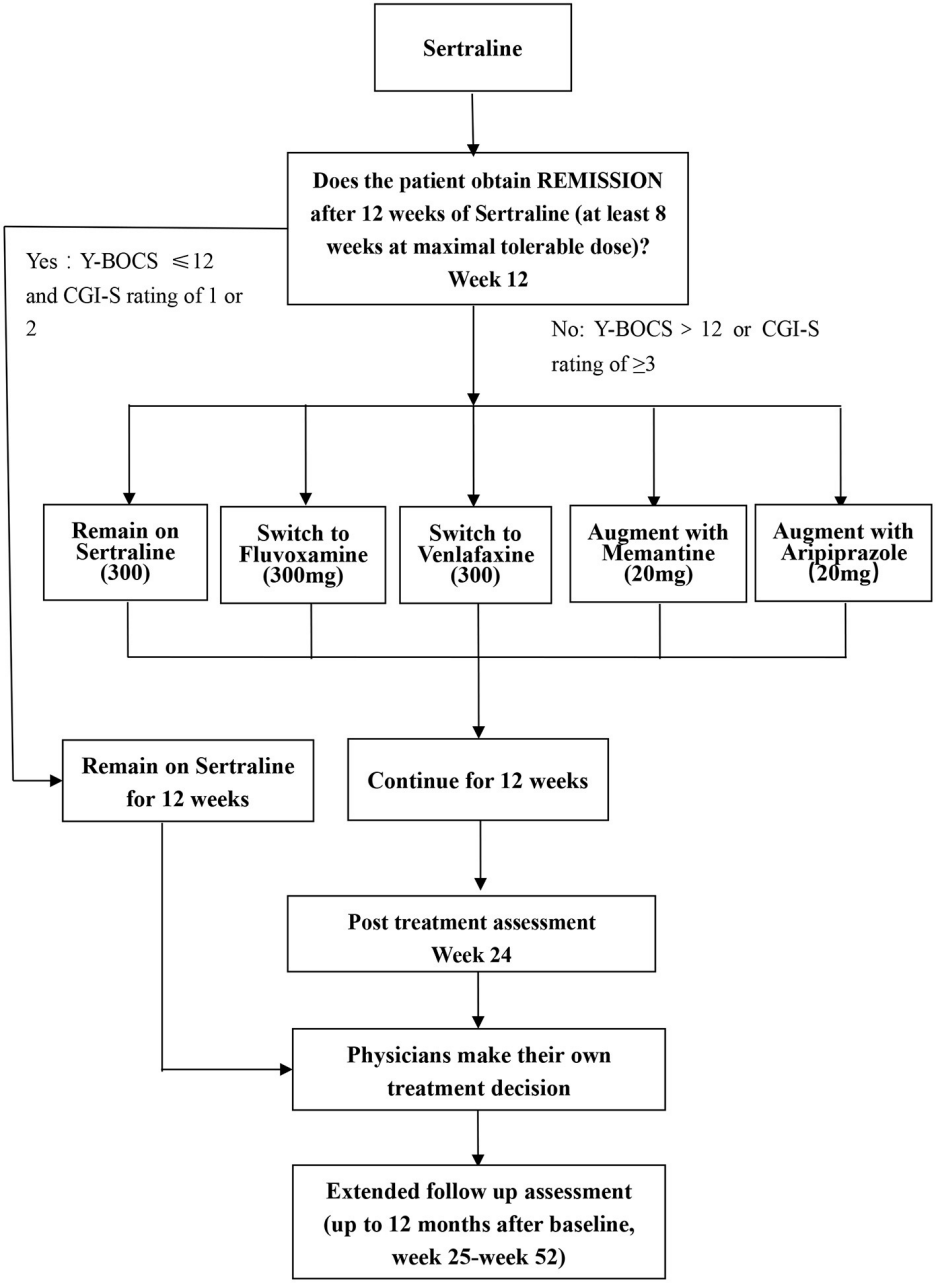
Flow chart of this study. Y-BOCS, Yale-Brown Obsessive Compulsive Scale; CGI, clinical global impression.

A randomized block design will be used in this study and all eligible participants accepted into this study will undergo an initial course of pharmacotherapy (phase I), and non-remitters will be randomly allocated to five treatment arms (phase II). In phase II, the 5 arms will comprise (1) treatment with higher than usual doses of sertraline, (2) switch to fluvoxamine, (3) switch to venlafaxine, (4) augmentation with memantine, and (5) augmentation with aripiprazole. Physician and patients will know which treatment arm is employed, but change in OCD symptoms will be assessed at several different time points by independent evaluators (IEs) who will be blind to treatment assignment. The flow diagram of the study protocol is shown in Figure 1.

### Inclusion/ Exclusion Criteria

Participants will be recruited from Shanghai Mental Health Center and twelve other specialized OCD sites in China. Individuals will be included in the study if they (1) meet the Diagnostic and Statistical Manual of Mental Disorder, fifth Edition (DSM-5) criteria for OCD as the primary diagnosis (17); (2) are in the age range from 18 to 65 years; (3) have a score of at least 20 on Yale-Brown Obsessive-Compulsive Scale (Y-BOCS) (18); (4) have never received medication for OCD, and have not received any form of psychotherapy for OCD in the past 6 month; (5) have provided written informed consent.

Participants will be excluded if they (1) have met the DSM-5 diagnostic criteria for Schizophrenia Spectrum and Other Psychotic Disorders, or the Bipolar and Related Disorders; (2) have a moderate or higher risk of suicide (?9 on the Suicide Module in the Mini-International Neuropsychiatric Interview (MINI) (19); (3) have substance use that is sufficiently severe to possibly impact negatively on treatment adherence in the past 1 year; (4) have severe depression with Beck Depression Inventory-II (BDI-II) score of ≥29 (20); (5) have comorbid psychiatric or medical disorders that may impact negatively on adherence to or on the efficacy of medication (e.g., borderline personality disorder, CNS disorders); (6)are pregnant or lactating females.

### Screening and Baseline Visit

All recruited patients will be diagnosed as having OCD as the primary diagnosis by a psychiatrist, using DSM-5 criteria. The Y-BOCS (18) will be used to assess the severity of OC symptoms, and the MINI (19) will be used to screen history of comorbid DSM-5 psychiatric disorders. At baseline, demographic data (including age, gender and education) will be collected, and participants will undergo a brief physical examination including blood routine and liver function tests. In addition, participants will complete the self-administered Obsessive-Compulsive Inventory Revised (OCI-R) (21), BDI-II, Beck Anxiety Inventory (BAI) (22) and Sheehan Disability Scale (SDS) (23) to measure the severity of clinical symptoms. The Chinese versions of all measures have been proved to be reliable and eligible (24–27).

### Interventions

#### Experimental Phase I: Initial Treatment

The protocol will be approved by the Institutional Review Board of all participating centers. All recruited participants will provide written informed consent before any study procedures are undertaken. Participants will be made fully aware of the design of the study, and of possible adverse effects of the medications under investigation.

Sertraline was chosen to represent the SSRIs as the initial treatment. The rationale for this chosen is as following: First, sertraline is one of four SSRIs approved by the National Medical Products Administration (NMPA) for the treatment of OCD in China, and is recommended as first-line agent by the Chinese Practice Guideline on OCD (9). Second, in terms of drug metabolism, sertraline demonstrates linear pharmacokinetics when daily dose is between 50 and 200 mg (28), so it is straightforward for physicians to adjust the dose according to the patient's condition. Third, there is good evidence of the safety and tolerability of sertraline, and this medication has few drug interactions. In addition, sertraline is considered the medication treatment of choice for OCD in the clinical practice in China.

In phase I, participants will receive sertraline, initially at 50 mg/d, with a weekly 50 mg/d further increase, to the maximum recommended dosage (200 mg/d) or to the maximum tolerated dosage (<200 mg/d). Patients will be on their maximum dose by week 4, so allowing an assessment of response at 12 weeks (29).

- If the patients achieved remission in the first-step treatment [scores of ≤12 on the Y-BOCS and Clinical Global Impression –Severity (CGI-S) rating of 1 or 2 at week 12], participants will be continued on sertraline for another 12 weeks (30).
- Participants who do not achieve remission in the first-step treatment (scores of >12 on the Y-BOCS or CGI-S ≥3) will be randomly assigned to the second-step treatment. However, participants who do not achieve remission because they cannot tolerate a high dose of sertraline (200 mg) will not enter the second phase.

#### Experimental Phase II: Sequenced Treatment Alternatives

The second-step therapy will consist of five treatment options including higher-than-usual-maximal dosage of sertraline, switching to fluvoxamine, switching to venlafaxine, augmentation with memantine, and augmentation with aripiprazole. The rationale for each of these treatment options follows.

- *Higher-than-usual-maximal dosage of sertraline:* Previous evidence from a meta-analysis of OCD pharmacotherapy suggested that higher doses of SSRI may allow a more optimal response with similar adverse effects, when compared with lower doses of SSRI (31). Furthermore, a multicenter double-blind randomized controlled trial revealed that a higher-than-usual dose of sertraline (250–400 mg/d) was more efficacious than a standard dose (200 mg/d), but with similar tolerability (32). In view of this literature, but also considering the precautionary principle, we set an intermediate dose (300 mg/d) as the upper limit.
- *Switching to fluvoxamine:* Research suggests that different SSRIs may have equal efficacy in OCD, but treatment guidelines note that different SSRIs may have slightly different pharmacological profiles and suggest that some patients who do not responds to a particular SSRI may respond to a different one (33). There is good evidence for the efficacy of fluvoxamine in OCD. In addition, fluvoxamine is considered the second medication treatment choice for OCD in China (34).
- *Switching to venlafaxine:* Although venlafaxine is not considered a first-line agent for OCD, this serotonin-norepinephrine reuptake inhibitor (SNRI) has a different pharmacological mechanism from the SSRIs, and so is recommended for patients who do not remit after treatment with an SSRI in a number of practice guidelines (6, 8). A randomized double-blind study also showed that venlafaxine (300 mg/d) was equally effective to paroxetine (60 mg/d) in treating patients with OCD (35). A real-world research also suggested that venlafaxine may be useful in a proportion of patients with poor response to SSRIs (36). In addition, the maximum dose of venlafaxine approved for clinical practice in China is 300 mg/d. So, we set the target dose at 300 mg/d although the recommendation for venlafaxine is 350 mg/d in some countries.
- *Augmentation with memantine or aripiprazole:* Augment treatment strategies have widely been recommended and studied in patients who have not had an adequate response to SSRIs. Randomized controlled trials (RCTs) and open-label data have both demonstrated that SSRI augmentation with memantine, a glutamatergic agent, is superior to placebo in OCD (37, 38). Similarly, RCTs and open-label data have demonstrated that SSRI augmentation with aripiprazole, an atypical antipsychotic, is efficacious in OCD (39). There is some evidence that aripiprazole has a superior efficacy and tolerability profile compared to other antipsychotic agents when used to augment SSRIs in OCD (39). Thus, a number of practice guidelines suggest that SSRI augmentation with memantine and aripiprazole may be considered in the management of OCD (6, 8, 40).

In summary, during phase II, the treatment options will be:

- Remain on sertraline (higher dosage): where sertraline 200 mg has been tolerated, dosage will be increased by 50 mg fortnightly to a maximal dose of 300 mg/d or to the maximum tolerable dose (<300 mg/d).
- Switch to fluvoxamine: fluvoxamine will be initiated at a dose of 50 mg/d, increasing quickly to a maximal dose of 300 mg/d or the maximum tolerated dose by week 4 (41).
- Switch to venlafaxine: venlafaxine will be initiated at 75 mg/d, increasingly weekly by 75 mg/day, to a maximal dose of 300 mg/d or the maximum tolerated dose (35).
- Augment with memantine: sertraline will be augmented with memantine initially at 5 mg/d, and increasing by 5 mg/d weekly to a maximal dose of 20 mg/d (10 mg twice daily) or the maximum tolerated dose (42).
- Augment with aripiprazole: sertraline will be augmented with aripiprazole, initially at 5 mg/d, and increasing by 5 mg/d weekly to a maximal dose of 20 mg/d or the maximum tolerated dose (43).

At the conclusion of phase II at week 24, physician may choose to continue the current treatment regime, or to make appropriate changes, as per the principles of the Chinese Practice Guideline on OCD (which are similar to those of other practice guidelines from around the world) (6–9). The extended follow up assessment will last up to the month 12 after baseline (i.e., week 25-week 52) if participants are willing to stay in the program.

Adjuvant therapy with benzodiazepines or non-benzodiazepines for insomnia will be allowed during the course of the study, but other psychotropic agents will not be permitted. Participants can withdraw from the study at any time if they wish to do so, without any consequences.

### Sample Size

To determine the detectable effect size, sequential multiple-assignment randomized clinical trials (SMART) were used to verify the design of pharmacotherapy options following unsatisfactory initial treatment in OCD. Given preliminary data and literature retrieval, an expected remission rate to Y-BOCS (phase I) (44). In phase II, there are 5 groups. Through multiple comparisons, the clinical remission rate of the two groups with the smallest difference was evaluated, P_T = 20%, P_C = 10%. We use the type I error at 0.05 (α = 0.05), type II error at 0.2 (β = 0.2) and get a sample size of 199 in each group [total sample size is 199^*^5/(1–20%) = 1,244] using PASS software version V11. Consider a 20% drop-out rate, the minimum sample size of *N* = 1,600 for Phase I was calculated according to the possible combination.

### Primary Outcome

The primary outcome will be OCD symptoms severity as measured by the Y-BOCS (18) and by IEs. The Y-BOCS has been shown to have good psychometric properties, is sensitive for measuring treatment effects, and is regarded as the gold standard for symptom severity assessment in trials of OCD.

### Secondary Outcome

The secondary outcomes will comprise clinical severity (anxiety, depression, and obsessive-compulsive symptoms), functional impairment and side effects at different time, as assessed with the following measures:

#### Clinical Global Impression

The Clinical Global Impression (CGI; National Institute of Mental Health) (45) is a clinician-rated scale to assess treatment response in patients with mental disorders. It requires the clinician to rate how much the patient's illness has improved or worsened relative to a baseline measurement (phase I: week 0; phase II: week 12).

#### Beck Anxiety Inventory

The Beck Anxiety Inventory (BAI) (22) is a 21-item, self-report inventory which identifies anxiety symptoms and quantifies their intensity. Respondents are asked to rate how much they have been bothered by each item over the past week.

#### Beck Depression Inventory-II

The Beck Depression Inventory-II (BDI-II) (46) is a 21-item, self-report rating inventory that measures characteristic attitudes and symptoms of depression. It includes both cognitive and somatic symptoms of depression.

#### Obsessive-Compulsive Inventory-Revised

The Obsessive-Compulsive Inventory Revised (OCI-R) (21) is a 18-item self-report measure of obsessive-compulsive measures. It is chosen because of its psychometric properties and the short time that its administration requires.

#### Sheehan Disability Scale

Sheehan Disability Scale (SDS) (23) is a reliable, brief self-report scale that assesses disability or functional impairment on 3 items: work, social life and family life.

#### Treatment Emergent Symptom Scale

The Treatment Emergent Symptom Scale (TESS) (47) is widely used to record side effects. We will use it to assess tolerability of the different treatment arms.

#### Tolerability

The tolerability of treatment will be defined as side effect discontinuation in this study. as defined by the proportion of patients who discontinued treatment due to adverse events during the study (48).

### Medication Management

Screening visit will include Informed consent, demographic data, MINI Psychiatric Interview, symptom measures. All the recruited participants of this study are interviewed and evaluated by IEs who are blind to randomization every two or four weeks after enrollment and following-up evaluation will be up to week 24. If participants are willing, we will continue to extend follow up interview for up to 12 months after baseline (week 25-week 52). Details are provided below (Table 1).

1. Interview 1 (week 0): verification of inclusion/exclusion criteria, assessment of insight, symptom measures, laboratory investigation (e.g., electrocardiogram, blood testing, and other necessary tests).
2. Interviews 2–8 (week 2, 4, 8, 12, 16, 24): follow up with symptoms measures, laboratory investigation, recording adverse events, ending record.
3. Extended interview (up to 12 months after week 0): follow up with symptom measures, laboratory inspection, recording adverse events.

**Table 1:** Timing of assessments and data collection.

	**Baseline**	**Treatment and follow-up phase**							**Extended follow up**
**Timepoint (Weeks)**	**0**	**2**	**4**	**8**	**12**	**16**	**20**	**24**	**25–52**
Informed consent	X								
MINI	X								
demographic data	X								
**Primary endpoint**									
Y-BOCS	X	X	X	X	X	X	X	X	X
**Secondary endpoint**									
OCI-R	X	X	X	X	X	X	X	X	X
BDI-II	X	X	X	X	X	X	X	X	X
BAI	X	X	X	X	X	X	X	X	X
SDS	X	X	X	X	X	X	X	X	X
CGI		X	X	X	X	X	X	X	X
TESS		X	X	X	X	X	X	X	X
Tolerability		X	X	X	X	X	X	X	X
Ending record								X	

*MINI, the Mini-International Neuropsychiatric Interview; Y-BOCS, Yale-Brown Obsessive Compulsive Scale; OCI-R, Obsessive-Compulsive Inventory Revised; BDI-II, Beck Depression Inventory-II; BAI, Beck Anxiety Inventory-; SDS, Sheehan Disability Scale; CGI, Clinical Global Impression; TESS, Treatment Emergent Symptom Scale. X represents must-done item*.

All participants will complete the self-reports mentioned above and will be administered the clinician-rated scales either face-to-face or via telephone or by remote video conferencing.

### Adherence

To increase adherence to pharmacotherapy, the clinician treating the patient will emphasize the importance of continuous administration of medication at each visit. In addition, all participants will be asked to record their daily medication intake in standard forms. Drug dosage reduction, increase or missing administration will be recorded. From all records the participants will be divided into three categories: good compliance, defined as medication taken as intended; moderate compliance, defined as reduced medication to one to two tablets per day for a maximum of 4 weeks and/or no tablets for a maximum 2 weeks as projected; and poor compliance, defined as reduced medication to one to two tablets per day more than 4 weeks and/or no tablets more than 2 weeks.

### Safety and Monitoring

The TESS will be used by the clinician treating the patients to evaluate side effects at each visit following initiation of treatment. Adverse and secondary events will be recorded from the baseline visit and throughout the follow-up visits. All adverse events will be report to the administration agency of the hospital. Serious adverse events will be reported to the Ethics Committee at each site and to the lead site. The association of an event with study drugs will be evaluated based on a temporal and biological correlation analyses.

### Data Collection and Management

Participants in this study will be interviewed and evaluated by IEs in the study, and the data will be directly collected using the electronic data management system.

- Electronic Case Report Form (eCRF): Data administrators will build eCRFs according to the research protocol.
- Permission Assignment: Data administrators will create accounts and grant different permissions to access the system according to different identities of researcher, rater and inspector. For example, researchers of each center will only be able to browse and modify the data they collect themselves. The case situation at each center will be read by the supervisor, who has no authority to modify the data, but who can comment or raise questions.
- Data input: Clinical researchers or coordinators will promptly and accurately enter the data into the eCRF.
- Data Questions and Answers: For the questions in the case report, the independent supervisor will issue the Q&A form, and the researcher or evaluator should answer and return it as soon as possible. The data administrator will modify the data according to the answers of the researcher and may issue the Q&A form again if necessary.
- Data Locking and Exporting: After each participant completes the experiment and is verified by the supervisor, the data manager will lock the data. During the experiment, real-time lock data was exported for interim analysis as required. After all the test data were locked, the data manager will export them to the designated database for final statistical analysis.

### Statistical Methods

Descriptive analysis and statistics will be undertaken with SPSS 20.0. All the relevant data of patients meeting the inclusion criteria will be used as the data set for analysis. Comparison among the clinical and demographic characteristics of the samples will conducted by chi-square test and *t*-test. The patient's adherence to treatment and the factors affecting prognosis will be classified and counted by SPSS combined with R. Ordinal multi-categorical logistic regression model will be used to construct the index system of symptom severity. Mixed-effect models will be used to evaluate the efficacy of treatment on both the primary treatment outcome measure (the YBOCS) as well as on secondary treatment outcome measures.

### Informed Consent

The researcher needs to explain in detail the purpose of this study, research contents, the potential risk and benefit, alternative therapies available and the rights and obligations of the participants in line with the Helsinki Declaration to all screening participants (compulsive disorder patients willing to participate in the study). The medical treatment of patients who do not agree to enter the study will not be impacted. Participants have the right to withdraw at any stage after they enter the study. The explanation should also include the necessary matters to protect the individual rights and interests of the participants. With full explanation, after confirming that the patient fully understands the study and clearly knows the informed consent, the participant can enter the research process only by signing the informed consent and indicating the date.

If the informed consent is revised during the study, the modified content of the informed consent shall have an impact on the patients out of the group after the end of the study, and patients undergoing treatment or follow-up shall sign the newly revised informed consent again.

### Quality Control

Research coordinator of each research center is responsible for the coordination in the research, and the implementation of the project schedule management. The independent supervisor will monitor the quality of the work by reviewing informed consents, and completion of eCRFs. They will visit site every 6 months. 25% of all cases are randomly selected for checking by the independent monitor. In addition, all sites will submit a brief description of the clinical features of their patients at time of recruitment to monitor accuracy of diagnosis (for example, excluding patients with body dysmorphic disorder). In addition, all IEs will receive online training in administration of the YBOCS and CGI. The training process will comprise introductory lectures and demonstrations by experienced raters, followed by repeated rating of videos until such time as inter-rater reliability is obtained. To maintain reliability of ratings, raters will receive additional training up to four times per year and inter-rater reliability will be assessed.

## Discussion

The current clinical trial is being conducted to compare different approaches to the pharmacological treatment of OCD patients who do not respond, or who respond but do not remit, to first line SSRI pharmacotherapy. To the best of our knowledge, this will be the first study to compare the efficacy of several switching or augment strategies for medications in such patients, and the largest prospective randomized pragmatic trial of OCD to date. Our hope is that this study will provide useful evidence to inform clinical practice and international guidelines on the pharmacological management of OCD.

There are several unique features of this study. First, the design is pragmatic in several respects, for example, patients with secondary major depression will be included, and physicians and patients will not be double-blind to treatment. Second, the design is rigorous, with randomization to different treatment arms, and with raters being kept blind to treatment randomization in Phase II. Third, the trial will compare several augmentation and switching strategies. Finally, this will be the largest RCT of OCD ever conducted to date.

In conclusion, this pragmatic study is the first randomized trial that compares 5 different pharmacotherapy options in patients who do not have remission after treatment with an SSRI, and it should provide data that are useful in informing the optimal pharmacotherapeutic approach to such patients. It hopes to complement the existing literature on large pragmatic trials of schizophrenia, bipolar disorder, and major depression, by contributing equally informative data on OCD.

## Trial Status

This trial is recruiting and is expected to be complete in December 2024.

## Ethics Statement

Ethical approval for this trial has been granted by the Institutional Review Boards of Shanghai Mental Health Center and other participating institutions 20/04/2020 (2020- 11), and written informed consent will be obtained from all participants. The trial is registered as NCT04539951.

## Author Contributions

Preparation of the original manuscript draft was conducted by PW and WG, with reviewing and editing by HS, DS, and ZW. JG, CW, JF, MH, HX, BL, NL, WT, XW, YJ, YL, YC, and ZT are responsible for study assessments and monitor the participants at 13 clinical centers. The design of the study was done jointly by all authors. All authors have read and agreed to the published version of the manuscript.

## Funding

This clinical trial was supported by the key programs of Shanghai Mental Health Center (CRC2018ZD03) and Shanghai Clinical Research Center for Mental Health (19MC1911100).

## Conflict of Interest

The authors declare that the research was conducted in the absence of any commercial or financial relationships that could be construed as a potential conflict of interest.

## Publisher's Note

All claims expressed in this article are solely those of the authors and do not necessarily represent those of their affiliated organizations, or those of the publisher, the editors and the reviewers. Any product that may be evaluated in this article, or claim that may be made by its manufacturer, is not guaranteed or endorsed by the publisher.
